# An Overview of the Role of Calcium/Calmodulin-Dependent Protein Kinase in Cardiorenal Syndrome

**DOI:** 10.3389/fphys.2020.00735

**Published:** 2020-07-14

**Authors:** Carolina Victoria Cruz Junho, Wellington Caio-Silva, Mayra Trentin-Sonoda, Marcela Sorelli Carneiro-Ramos

**Affiliations:** ^1^Center of Natural and Human Sciences (CCNH), Universidade Federal do ABC, Santo André, Brazil; ^2^Division of Nephrology, Department of Medicine, Kidney Research Centre, Ottawa Hospital Research Institute, University of Ottawa, Ottawa, ON, Canada

**Keywords:** CaMKII, cardiorenal syndrome, inflammation, immune system, cardiovascular diseases

## Abstract

Calcium/calmodulin-dependent protein kinases (CaMKs) are key regulators of calcium signaling in health and disease. CaMKII is the most abundant isoform in the heart; although classically described as a regulator of excitation–contraction coupling, recent studies show that it can also mediate inflammation in cardiovascular diseases (CVDs). Among CVDs, cardiorenal syndrome (CRS) represents a pressing issue to be addressed, considering the growing incidence of kidney diseases worldwide. In this review, we aimed to discuss the role of CaMK as an inflammatory mediator in heart and kidney interaction by conducting an extensive literature review using the database PubMed. Here, we summarize the role and regulating mechanisms of CaMKII present in several quality studies, providing a better understanding for future investigations of CamKII in CVDs. Surprisingly, despite the obvious importance of CaMKII in the heart, very little is known about CaMKII in CRS. In conclusion, more studies are necessary to further understand the role of CaMKII in CRS.

## General Considerations

Calmodulin (CaM) is a low-molecular-weight protein highly conserved in the eukaryotes (Clapham, 2007). CaM was discovered in 1970 as a calcium (Ca^2+^) regulator in the brain, responsible for the nucleotide phosphodiesterase. It was first mentioned as a Ca^2+^-dependent regulator (Kakiuchi and Yamazaki, 1970). Since the origin of eukaryotes, the amino acids that compound CaM have not changed at all (Friedberg and Rhoads, 2001). It plays a fundamental role in every cell by amplification of the Ca^2+^ signal (Clapham, 2007). Ca^2+^ is a versatile messenger molecule implied in many basic processes, such as contraction, potentiation, cell proliferation and apoptosis, and others (Carafoli and Krebs, 2016). To maintain a homeostasis of Ca^2+^ in the intracellular environment, it has many mechanisms and signaling paths that help to establish a gradient of this ion, holding at approximately 2 mM (Clapham, 2007). One of them is the CaM that triggers conformational changes in response to Ca^2+^ oscillations (Carafoli and Krebs, 2016). In other words, when Ca^2+^ binds to CaM, it induces a structural modification, forming a Ca^2+^/CaM complex (Clapham, 2007).

There is a vast quantity of proteins that can bind to Ca^2+^/CaM via an α-helical region. This region is composed of approximately 20 amino acids, positively charged and containing hydrophobic residues (Islam, 2020). To amplify the signal generated by Ca^2+^, this complex activates or deactivates phosphorylation pathways, targeting a protein kinase dependent on Ca^2+^/CaM (CaMK) (Clapham, 2007). In other words, the binding of Ca^2+^/CaM and phosphorylate serine/threonine residues of target proteins are the main trigger of CaMKs activation, initiating signaling activation of the substrates (Takemoto-Kimura et al., 2017).

The Ca^2+^/CaM-stimulated protein kinases are divided based their substrate specificity. They can be restricted to a small number of substrates (Islam, 2020) while the multifunctional kinases have wide specificity and regulate multiple functions in the same and different cell types (Skelding et al., 2011). Restricted CaMK have three main families: phosphorylase kinase (PhK), elongation factor 2 kinase (eEF2K), and myosin light chain kinase (MLCK) (Skelding and Rostas, 2012). These families do not share common domains or structures, making them more specific to certain stimuli and pathways. On the other hand, the multifunctional kinases control many cell functions in different cell types, which makes them a powerful controller of other kinases. Regulation of Ca^2+^ dynamics is the most basal method to control the function of a kinase, mainly intracellular concentration of Ca^2+^ (Skelding and Rostas, 2012). The main multifunctional families of kinases proteins are CaMKI, CaMKIV, CaMKK, and CaMKII (Hudmon and Schulman, 2002).

The CaMKI family constitutes four elements, each of them encoding a different gene: CAMK1 (CaMKIα), PNCK (CaMKIβ/Pnck), CAMK1G (CaMKIγ/CLICK3), and CAMK1D (CaMKIδ/CKLiK), which are found in higher quantity in mice brains (Picciotto et al., 1995). CaMKI function is observed in many cellular activities, including synapsis in terminal nerves, motility, axon growth, synthesis of aldosterone, and the cell cycle (Condon et al., 2002; Skelding et al., 2011). It is known that CaMKI translocates to the nucleus mediated by CRM1 complex after an influx of intracellular Ca^2+^ induced by potassium depolarization or glutamate (Sakagami et al., 2005). CaMKIV only encodes one gene, CAMK4, coding the monomerics isofroms: α and β (Skelding and Rostas, 2020). CaMKIV is observed in the regulation of cyclic AMP, plasticity, fear memory, inflammatory sensibility, and control of the cell cycle (Skelding and Rostas, 2020). The CaMKIV can translocate between the cytoplasm and nucleus. For that action, it involves some catalytic activity as catalytically inactive CaMKIV remains in the cytoplasm (Lemrow et al., 2004).

Ca^2+^/CaM-stimulated protein kinase kinase (CaMKK), CAMKK1 and CAMKK2, produce CaMKKα and CaMKKβ, respectively. They are responsible for many functions (Skelding and Rostas, 2020). Studies show this kinase’s function, suggesting that CaMKK translocates to the nucleus under stimulation, and the inhibition of translocation directly implies the deactivation of monocytic cells (Guest et al., 2008). CaMKI, CaMKIV, and CaMKK share the same signaling pathway. It is called the Ca^2+^/CaM-dependent kinase cascade (Tokumitsu and Soderling, 1996). This pathway is mainly related to several cellular processes, including glucose homeostasis, hematopoietic stem cell maintenance, cell proliferation, apoptosis, and normal immune cell function (Skelding and Rostas, 2012). Last but not least, among the CaMKs, we have the CaMKII. Its function is further explained in the next section. Together with this, the present study aims to focus on CaMKII relevance and how it is implied specifically during cardiorenal syndrome (CRS).

## The Calcium-Calmodulin-CAMKII Signaling Axis Has a Crucial Role in Cardiac Function

Among the CaMKs, the most abundant in the heart is CaMKII (Luczak and Anderson, 2014). CaMKII is a serine-threonine kinase, and it was first identified in the central nervous system, where it represents only 2% of the total protein. It was later discovered that this enzyme is present in many tissues, including the pancreas, where it has an important role in the secretion of insulin, and in heart tissue, where it is responsible for Ca^2+^ homeostasis (Yamauchi, 2005).

**FIGURE 1:**
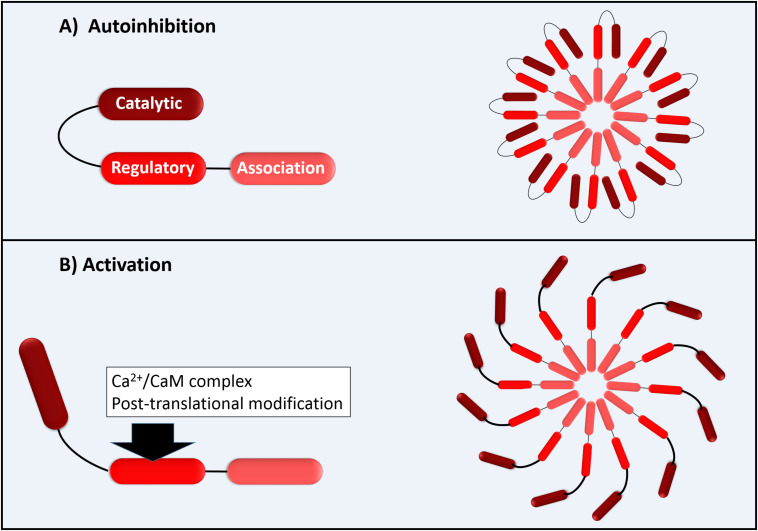
Schematic illustration of CaMKII with permission and adapted from Bussey and Erickson (2018). **(A)** Autoinhibition of each 12 CaMKII monomers; **(B)** Activation of CaMK monomers by Ca^2+^/CaM complex (direct activation) or by post-translational modifications as by ROS (autonomous activation).

The functional CaMKII enzyme structure is formed by 12 subunits (dodecameric); each monomer has an N-terminal catalytic domain and a C-terminal domain with a regulatory domain in the middle as shown in Figure 1. The catalytic domain is blocked by the regulatory domain in an autoinhibitory way, keeping the enzyme inactive (Figure 1). CaMKII becomes active when the Ca^2+^/CaM complex binds to the binding site of the regulatory domain of CaM. This provokes a successive autophosphorylation of Thr287 monomers, causing conformational changes that expose the catalytic domain and enable the kinase activity at all (Beckendorf et al., 2018). As proposed by Beckendorf et al. (2018), the Thr287 autophosphorylation causes what they denominate “CaM trapping,” increasing the CaM binding affinity to 1000-fold, maintaining the CaMKII activity even under low Ca^2+^ conditions (Beckendorf et al., 2018). Besides the activation via Ca^2+^/CaM that is dependent on several Ca^2+^ factors, such as the total Ca^2+^ available in a dose-dependent manner and its spark frequency, amplitude, and duration, the CaMKII can be activated via post-translational modifications as, for example, by reactive oxygen species (ROS) (Beckendorf et al., 2018).

CaMKII is observed in eukaryotes in four distinct isoforms (α, β, γ, δ). They encode four distinct genes (Luczak and Anderson, 2014). CaMKIIα and β are expressed mainly in the neural system while the CaMKIIδ and γ isoforms are predominantly expressed in cardiac tissue (Backs et al., 2009). This kinase modulates numerous biological processes, such as the Ca^2+^ homoeostasis, excitement of membrane, cell cycle, cytoskeletal organization, and gene expression (Yamauchi, 2005; Backs et al., 2009).

As the contraction is a Ca^2+^-dependent process, it is indisputable that CaMKII performs an important role in the heart. It regulates Ca^2+^-handling proteins by facilitating the L-type Ca^2+^ channel (LTCC) phosphorylation (Bers and Morotti, 2014); promotes phosphorylation of phospholamban (PLN) at site T17, promoting its dissociation from SERCA2a and, thus increasing SERCA2a activity; and phosphorylates ryanodine (RYR) at site S2814, consequently improving its opening probability (Mattiazzi and Kranias, 2014). Hyperphosphorylation caused by CaMKII of this site leads to an increase of spontaneous elementary Ca^2+^-release events from the sarcoplasmic reticulum especially during diastole (Mustroph et al., 2017). In pathological situations, increased diastolic Ca^2+^ in cytosol can activate the electrogenic NCX, which can cause delayed afterdepolarizations. The last ones can trigger atrial and ventricular arrhythmias (Mustroph et al., 2017). In other words, CaMKII is a pro-arrhythmogenic protein in the heart. Besides arrhythmias, CaMKII is strongly increased during myocardial injury (Ren et al., 2003), atrial fibrillation (Liu et al., 2019), cardiac hypertrophy (Kamada et al., 2019), ischemia/reperfusion injury (Rajtik et al., 2016), and heart failure (HF) (Beckendorf et al., 2018). Its inhibition has been constantly suggested as treatment for these pathologies (Rokita and Anderson, 2012; Cipolletta et al., 2015).

There are studies using transgenic (TG) overexpression of CaMKIIδ, to evaluate its effect during pathogenesis. TG mice developed severe HF and susceptibility to induced ventricular arrhythmias via programmed electrical stimulation (Maier and Bers, 2007). The cardiomyocytes isolated from TG mice presented increased diastolic Ca^2+^ leak together with prolonged action potential and increased incidence of early afterdepolarization (a peculiarity of CaMK-induced increased late I_Na_) (Wagner et al., 2006).

In summary, TG mice led to a worsening of the arrhythmogenic profile of the mice in addition to increasing their risk of life. On the other hand, the silencing of CaMKII brings hope for the treatment of chronic or acute heart diseases, presenting itself as a potential clinical therapist against these pathologies.

## The Role of CAMKII in Cardiorenal Syndrome

The kidneys and the heart share an important role in the biochemical maintenance of homeostatic function of extracellular fluid. In general, while the heart is responsible for providing nutrients and oxygen-rich fluids to the body through blood flow, the kidney is responsible for providing electrolytes, acid-base homeostasis, vitamin D activation, and erythropoietin synthesis (Ronco and Di Lullo, 2014). Understanding how the kidney and heart relate has been a challenge since the Middle Ages, when Aetius of Amida initially attempted to explain fluid overlay by attributing it to kidney hardening (Diamandopoulos, 1999).

Fast-forwarding to contemporary times, several studies approach this topic with more precision. Nowadays, it is well established that an adverse imbalance in hemodynamics caused by impaired kidney function can directly impact the heart (Anavekar et al., 2004). The pathological crosstalk between both organs is called cardiorenal syndrome (CRS), in which cardiac and renal dysfunction overlap; a disorder in one organ leads to acute or chronic dysfunction of the other organ (Ronco, 2011; Ronco and Di Lullo, 2014).

CRS is divided into two major groups, the cardiorenal and nephron-cardiac, dependent on the origin of the primary pathology, both of which can be acute or chronic (Ronco and Di Lullo, 2014). Type 1 and 2 CRS are considered cardiorenal and are characterized by a loss of cardiac function leading to renal injury (Damman et al., 2007). CRS type 1, known as acute, is described by acute cardiac injury leading a renal one through hemodynamic mechanisms (Di Lullo et al., 2017). The presence of acute decompensated HF leads to decreased renal function due to low renal arterial flow and decrease of the glomerular filtration rate. Once hemodynamic parameters are restored, renal and cardiac homeostasis is also restored (Hanada et al., 2012). Type 2 CRS rises as a consequence of chronic abnormalities in cardiac function that cause renal injury or dysfunction. Examples of such abnormalities are conditions including atrial fibrillation, congenital heart disease, pericarditis constrictions, and chronic cardiac ischemia. Determining whether the CRS is a type 1 or 2 represents a challenge for clinicians since the majority of diagnostics are made when both organs are already injured.

The other two types of CRS (3 and 4) are described as nephron-cardiac syndromes, where the renal injury leads to cardiac dysfunction (Damman et al., 2007). Type 3 CRS is defined as acute nephron-cardiac syndrome, occurring when acute renal failure leads to the development of acute cardiac injury. It is intimately related to events triggering increased inflammatory processes, such as oxidative stress and secretion of neurohormones (Di Lullo et al., 2017). Type 4 CRS is studied as chronic nephron-cardiac disease, initiated by chronic kidney disease (CKD), leading to cardiovascular disease. Approximately 70–80% of patients with end-stage renal disease present type 4 CRS, presenting cardiac complications, such as infarct and long-term arrhythmias (Di Lullo et al., 2017).

Finally, CRS type 5 is a systemic disorder that reaches both organs simultaneously. Many factors have been suggested to contribute to these conditions, for instance, sepsis, infections, drugs, toxins, and diabetes. It is important to note that acute type 5 CRS can overlap a chronic injury (Ronco and Di Lullo, 2014). CRS type 5 results in cardiac and renal dysfunction coming from a larger and systematic situation. Hence, differently from other types of CRS, type 5 is relatively easier to identify the starting point of the CRS.

The study of CRS is of great relevance for clinical treatment, considering that cardiovascular diseases represent the main cause of death in the United States for at least the last 15 years, according to the Centers for Disease Control and Prevention (CDC)^1^. In addition, incidence of CVDs have been increasing alongside incidence of CKDs worldwide (Thomas et al., 2017), showing the importance of studies regarding CRS type 4, for example. Besides, CKDs significantly affects the regulation of cardiac Ca^2+^ by mechanisms not yet clarified (Ke et al., 2020).

CaMKII has already been described as a cause of many heart dysfunctions, such as arrhythmia, hypertrophy, and infarction (Yoo et al., 2018; Rusciano et al., 2019), and has been demonstrated to play a critical role in CRS types 1 and 2. The CaMKII inhibitor (CaMK2n) has been shown to protect cardiac dysfunction and ameliorate injuries observed in metabolic syndrome (Prasad et al., 2015). However, Alfazema and collaborators showed, recently, in a translational study, that deletion of CaMK2n1, diminishes CaMKII activity in the kidney and heart without affecting adipose tissue (Alfazema et al., 2019). Yet there are few studies involving both organs in a systemic profile.

It is known that elements, such as the immune system, can mediate the communication between them. In an inflammatory process as observed during CKD and chronic heart failure (CHF), cytokines are released by circulating and tissue-resident inflammatory cells (monocytes mainly) and play an important role in the progression of these diseases (Yogasundaram et al., 2019). Since Ca^2+^ has been associated with several events of the inflammatory response, including the activation of T cells and awakening memory (Boubali et al., 2012). The CaMKII ability to act as an intracellular sensor of Ca^2+^ makes it a crucial regulator in the inflammatory process (Rusciano et al., 2019). On the other hand, the Ca^2+^-independent form of CaMKIIγ also modulates cell death and T cell memory formation (Bui et al., 2000). Studies have already shown that CaMKII is capable of modulating NF-κB, IL-10, IL-2, and IL-4 production (Rusciano et al., 2019).

In CRS, the tissue injury is strongly followed by inflammation. Many inflammatory cytokines are enhanced in experimental models of renal ischemia (TNF-a, IL-1, and IL-6) and also markers, including the factor nuclear kappa B (NF-kB), which is very important for cell signaling during inflammatory processes (Trentin-Sonoda et al., 2015). Given that, some studies present the participation of inflammation as a cause of CKD progression in CHF patients (House et al., 2019; Marsico et al., 2019). During cardiac ischemia, myocytes release inflammatory cytokines (Colombo et al., 2012), and they can reach renal tissue, inducing local inflammation, apoptosis, or oxidative stress (Ronco and Di Lullo, 2014).

Colombo et al. (2012) suggest that inflammation during CRS is controlled by positive feedback mechanisms. Inflammation initiates vascular dysfunction, reducing the myocardial contractility and increasing myocardial cell death, linking CRS to apoptosis (Virzì et al., 2015b). Inflammation also causes progressive renal dysfunction and fibrosis, which continues to injure the organ, maintaining the cycle. Additionally, inflammation leads to the release of renin, activating the renin-angiotensin-aldosterone system (RAAS), which activates the sympathetic nervous system (SNS) by increasing serum norepinephrine concentrations and is the cause of ROS release from the inflammatory cells (Bongartz et al., 2005).

A previous study suggests that the toll-like receptor (TLR) pathway is linked with the activation of CaMKII in a model of myocardial infarction (Singh et al., 2012). Recently, Wu and collaborators demonstrated that miR-148a attenuates ischemia/reperfusion injury in liver, once CaMKIIα represses the TLR4 signaling pathway in vivo and in vitro, decreasing the production of pro-inflammatory factors (Zheng et al., 2018). Moreover, activation of CaMKII in macrophages is initiated by a significant trigger elevation of intracellular Ca^2+^. This progresses to the activation of myeloid differentiation factor 88 (MyD88)- dependent and Toll/interleukin-1 receptor domain, prompting proinflammatory factors. Ca^2+^/CaMKII is fundamental for macrophage response once it requires the complete activation of the TLR pathway (Liu X. et al., 2008).

As mentioned above and summarized by Rusciano et al. (2019) there is evidence that suggests the important role of CaMKII in many cardiac pathologies involving inflammation due its ability to enhance pro-inflammatory signaling and its responsiveness to inflammation by dysregulating the Ca^2+^ balance. In the kidneys, CaMKIIβ expression is associated to aldosterone-induced fibrosis (Park et al., 2018; Zhang et al., 2018) and also shows that the increase in mitochondrial fragmentation observed in hyperglycemia stress-mediated renal damage is due to the JNK-CaMKII-Fis1 pathway (Zhang et al., 2018).

### CamKII Inhibition Displays Cardioprotection

As discussed above, silencing CaMKII has great therapeutic value. There are many blockers and inhibitors used nowadays in research. The most known are KN-93 and AC3-I.

The first inhibitor described was KN-62 in 1990 (Tokumitsu et al., 1990). One year later, in 1991, Sumi M and collaborators described a new and more selective inhibitor called KN-93 (Sumi et al., 1991). They act by blocking the enveloping of Ca^2+^/CaM around the CaM-binding segment and automatically freeing this segment from the catalytic domain (Pellicena and Schulman, 2014). In other words, it is a CaM-competitive CaMKII inhibitor. It was already discovered that KN-93 can directly block the potassium current I_Kr_ and potassium voltaged channels, preventing arrhythmic properties of CamKII (Mustroph et al., 2017). An important item to bring up is that KN-62/93 binds to the holoenzyme and directly steps in the interaction of Ca^2+^/CaM but does not directly bind to CaM (Sumi et al., 1991). Besides belonging to a family of CaM antagonists, KN-93 cannot avoid the activity of autophosphorylation of CaMKII (Wong et al., 2019).

Some studies have proven the efficiency of KN-93 in heart pathologies in several animal models. Under in vitro and in vivo stimulations with isoproterenol, arrhythmias have been abolished after using NK-93 (Sag et al., 2011). It also prevents arrhythmias after models of acidosis, DOX-induced, NO-donor SNAP (Mustroph et al., 2017). On some models, the KN-93 does not seem to prevent but to slow the arrhythmia (as longer cycle length) without marked alterations in baseline ECG characteristics (Hoeker et al., 2016). KN-93 also reduces diastolic cytosolic [Ca^2+^] after induced HF (Sag et al., 2011). It is also an attenuator of Ca^2+^-leak in a diabetes model of GlcNAcase inhibition (Erickson et al., 2013) and myocardial hypertrophy. Another study shows the high capacity to inhibit the binding of CaM with Na_V_1.5, increasing the calcium release from RYR2 in cardiomyocytes independent from CamKII (Johnson et al., 2019). This suggests that KN-93 has interactions with the CaM-Ca^2+^ binding; however, to inhibit CaMKII specifically, more affinized compound is necessary.

Experiments using TG RYR-mutant mice (S2814D mutant) are naturally more susceptible to atrial fibrillation, and because of this, it is used as a well-established model. Atrial fibrillation, phospholamban phosphorylation, and diastolic Ca^2+^-leak are reduced after prior injection of KN-93 in these TG mice (Voigt et al., 2012). Cardiomyocytes isolated from TG CaMKIIδc knockout mice completely recover the contraction ability after acidosis injury (Mustroph et al., 2017).

There is also a highly specific inhibitor of CaMKII called autocamtide-3-derived inhibitory peptide (AC3-I). It is the most used one in studies that require the blocking of CaMKII, and it is resistant even to proteolysis. As mentioned, AC3-I is derived from autocamtide-3. The last one is a substrate for CaMKII and acts mainly in the Thr-9 phosphorylation site substituted with Ala (Maier and Bers, 2007). As the research regarding CaMKII increases, its therapeutic use implicated in pharmaceuticals has been studied more. Several studies, using these inhibitors mentioned above, impart a cardio protection.

As mentioned, the AC3-I blocker, is a highly specific inhibitor of CaMKII. This is the main reason it is used more in the studies concerning the participation of CaMKII in many cell functions. It can inhibit CAMKII more selectively than CaMKIV (more and a hundredfold). Studies using this blocker also imply cardio protection: preventing hypertrophy, reducing ventricular arrhythmias, improving mechanical function, reducing RyR2 lacking, and decreasing mortality of diabetic mice (Sag et al., 2011).

Some studies have proven the efficiency of AC3-I in pathologies in some animal models. As cited with KN-93, the AC3-I also reduces atrial fibrillation in TG RYR knockout mice (Chelu et al., 2009) and seems to protect the heart from the same atrial fibrillation in Ang II models *in vivo* and *in vitro* (Purohit et al., 2013). There is a study from our group using interference RNA (RNAi) to block the expression of CaMKIIδ. It demonstrates that CaMKIIδ is fundamental for cardiomyocyte hypertrophy; once blocking the expression, the LPS-induced hypertrophy is reverted (Cruz Junho et al., 2019).

There are several models of heart problems leading to CaMKII. CRS, already cited, seems to be one of them. Both kidneys and heart share many mechanisms of homeostasis, and any injury to one can lead to one in the other. It is known that CaMKII is increased in many models of heart injury, and some models of CRS can cause arrhythmias and AP chances as well as contraction irregularities (Navarro-García et al., 2018; Alarcon et al., 2019). CaMKII could be crucial to the progression of CRS, more specifically related to the progression from acute HF to chronic (types 1 and 2). In this scenario, the inhibition of CaMKII would be cardioprotective.

## Oxidative Stress and Epigenetics Factors as CAMKII Regulators

The close relation between inflammation and oxidative stress in pathophysiological processes also makes the balance between oxidant and antioxidant forces and, therefore, oxidative stress, one of the most important mechanisms as has been demonstrated in heart and kidney injury studies (Li et al., 2017; Oliveira et al., 2017; Liu and Liu, 2018; Songbo et al., 2019).

Even though physiological levels of oxidative species are necessary for cellular function, the oxidative stress caused by the overproduction of these molecules in both organs leads to a series of structural abnormalities via immune system activation and fibrotic promotion (Virzì et al., 2015a). Some of these pathologies include left ventricle hypertrophy, atherosclerosis, endothelial dysfunction, and fibrosis in the heart while in the kidney ROS promotes interstitial fibrosis and increased inflammation (Kumar et al., 2019). A study involving patients with CRS type 3 shows that they have an increased level of inflammatory and oxidative stress factors, including IL-6, myeloperoxidase, nitric oxide (NO), copper/zinc superoxide dismutase (SOD), and endogenous peroxidase (Virzì et al., 2015a). Oxidative stress triggers an inflammatory response, and this response induces more oxidative stress. This stress may be maintaining the previously mentioned cycle of damage. While Ca^2+^ is associated with inflammation, studies have shown a connection between oxidative stress and CaMKII activation (Erickson et al., 2008). Erickson et al. (2008) also demonstrate a dynamic mechanism for CaMKII activation, which occurs via oxidation of the methionine residue site on the CaMKII regulatory domain; this oxidation-dependent CaMKII activation is important to Ang II and apoptosis since CaMKII remains active after ROS oxidation even in the absence of the Ca^2+/^CaM complex. Some proteins maintain a redox sensor that regulates the cell response to oxidative stress (Kim et al., 2014). CaM is one of these proteins, and this oxidation leads to a regulatory cascade response with specific targets, including CaMKII (Snijder et al., 2011), plasma membrane Ca^2+^ (Anbanandam et al., 2005), and nitric oxide synthase (NOS) (Montgomery et al., 2003). As mentioned above, one of the mechano-chemotransductions that ROS induces Ca^2+^ release by CaMKII involves NOS (Jian et al., 2014). The endothelial dysfunction caused by oxidative stress leads to uncoupling of endothelial NOS (eNOS), leading to the production of more ROS (Münzel et al., 2017). In pathological conditions, such as inflammation, the vasculature expresses the inducible form of NOS (iNOS) (Münzel et al., 2017). This isoform of NOS produces an excessive amount of NO that mediates impaired vasoconstriction, which may be further worsened by the decreased of eNOS activity (Jian et al., 2014). The continuous exposure of NO induced by pro-inflammatory mediators inhibits endothelium-dependent relaxation by impairing the via CaMKII-dependent activation of eNOS (Kassler et al., 1997). In addition, studies have shown the role of NOS in the kidney, demonstrating that, when NOS activity is compromised, there are a series of renal dysfunctions that reduce glomerular perfusion and filtration, which may lead to a progressive scenario of hypertension and kidney injuries (Carlstrom and Montenegro, 2019).

Oxidative stress in CaMKII by methionine-oxidized CaMKII was also observed in patients with atrial fibrillation (Purohit et al., 2013; Yoo et al., 2018), which also demonstrates that oxidative stress can act in part through increased constitutive activity of CaMKII, creating a highly vulnerable substrate within the HF that promotes atrial fibrillation beyond fibrosis. On the other hand, Kong et al. (2018) shows that the oxidative stress in mitochondria can be reduced after the inhibition of CaMKII, alleviating the myocardial ischemia-reperfusion injury. In addition to the redox balance, other factors indirectly contribute to cardiac and renal alterations. Many approaches have been studied in order to set a start point for the CRS. One of them is epigenetics factors.

Epigenetics is the area of biology that studies changes in the functioning of a gene that is not caused by alterations in the DNA sequence and that perpetuate in the meiotic or mitotic cell divisions (Wu and Morris, 2001). Epigenetic modifications are highly coordinated processes of change that are not restricted to a specific phase of life. These characteristics are fundamental to diseases acquired throughout life. Epigenetic changes are divided into DNA methylation, histone modification, and noncoding RNA expression (Liu L. et al., 2008).

Studies have been developed to innovate the way to prevent CRS. Slowly, epigenetics is gaining space, and traditional mechanisms (such as RAAS and inflammation) are being replaced by other patterns of findings and prevention. Imaging the scale of modifications and mutations in a syndrome such as CRS, numerous cell lines may be altered and reprogrammed, in both heart and kidney. Studies have pointed out the role of epigenetics in the development of CRS (Gaikwad et al., 2010). In types 3 and 4, for example, renal failure increases cardiac histone H3 epigenetics, evidencing the crosstalk between renal failure and the transcription of cardiomyopathy-related genes (Gaikwad et al., 2010). It is important to mention that epigenetics in CRS itself are little studied when compared to the traditional mechanisms even though it is very promising. The focus of studies is linked to inflammation and oxidative stress, which we know to be the consequences of CRS. Abnormal defects in DNA methylation, histone modifications, and microRNA (miR) participate in renal injury (Beckerman et al., 2014); however, none of them are related to post-progression of HF. During HF independent of renal injury, we can note the expression of transcription factors, angiogenic factors, and natriuretic factors, often used as biomarkers of this condition. Epigenetic modifications regulate them. Pathological hypertrophy and compromised contractility are described to increase DNA methylation levels. Inhibition of DNA methylation has already been suggested as treatment for CHF (Yang et al., 2015); however, it should be carefully studied once the DNA methylation is comprehended.

Ca^2+^ signaling is involved in epigenetic regulation, and the study of its signaling can contribute to the development of new therapeutic strategies (Awad et al., 2015; Puri, 2020). CaMKIIδ has been already described as fundamental for cardiac hypertrophy development (Cruz Junho et al., 2019). This mechanism occurs after CaMKIIδ selectively phosphorylates HDAC4. During cardiac hypertrophy, there is an activation of fetal cardiac genes, an important mechanism regulated by CaMKIIδ-mediated H3 chromatin regulation. With the recent development of epigenetic studies, the use of ChIP-seq to evaluate H3 phosphorylation and binding of CaMKIIδ across the genome enables a profound knowledge of the genome-wide functional effect of H3 alteration by CaMKIIδ (Awad et al., 2015). This connection can lead to CRS types 1 and 2 and can be strongly linked to the diagnosis of heart dysfunction. In addition, it is extremely important to highlight the role of micro RNA (miR). miR is a short and noncoding RNA that interacts with the 3-untranslated region (UTR) of mRNAs blocking gene expression, degrading mRNAs and regulating protein expression (Winter et al., 2009; Krol et al., 2010; Qi et al., 2019; Yang et al., 2019).

To identify signaling pathways, there is a serviceable tool called gene set analysis (GSA). It uses statistical analysis to predefine gene sets involved in a specific cellular process. Thus, GSA is especially useful to infer functions of different miRNAs. For example, miR-185 has a key role during cardiac hypertrophy. This miRNA targets pro-hypertrophic genes, such as RhoA, Cdc42, and Stim1 in the heart. Given that, miR-185 is also a potent therapeutic target for cardiac diseases (Brown et al., 2006; Carè et al., 2007; Liu et al., 2011; Zhang et al., 2015). In addition, Kim et al. (2015) show that miR-185 not only acts as the genes mentioned, but also targeting genes involved in Ca^2+^-associated pathological hypertrophy, including CamKII. It is worth mentioning the role of miR-1, once alterations in its expression or inhibition have been discovered in many cardiac pathologies (Yang et al., 2007; Lu et al., 2009).

Recently, Zhang et al. proposed a possible mechanism by which *Lycium barbarum* polysaccharides (LBP) restore cardiac contractility induced by miR-1 overexpression. The authors show that LBPs prevent the reduction of CaM and cardiac myosin light chain kinase and their corresponding downstream proteins, including CaMKII due to miR-1 overexpression (Zhang et al., 2018).

Regarding inflammatory processes, miR-625-5p has been illustrated to inhibit inflammatory response in human bronchial epithelial cells and is downregulated in heart diseases once miR-625-5p is able to inhibit STAT3 and reduce the expression of CaMKII. Moreover, miR-625-5p attenuated Ang II-induced cardiac hypertrophy through CaMKII/STAT3 (Qian et al., 2019).

In relation to kidney disease, Park et al. (2018) shows that miR-34c-5p and CaMKII are involved in aldosterone-induced fibrosis in the kidneys. In addition, recent studies have focused on MiR regulation and exosomes, specialized nanosized membranous vesicles, in different experimental models. These membrane-bound vesicles (30–100 nm) are released from different cell types and deliver bioactive molecules, including microRNAs (miRs). Recently, literature demonstrated the regulation of oxidative stress in cardiac stem cells through the miR-214/CaMKII pathway after using exosomes derived from miR-214-enriched bone marrow-derived mesenchymal cells (Wang et al., 2018).

In addition to miRs, the role of long noncoding RNAs (lncRNAs) is well known. lncRNAs are transcribed RNA molecules >200 nucleotides in length without known protein-coding function, regulating gene expression at epigenetic, transcriptional, and post-transcriptional levels (Mercer et al., 2009). Previous studies report that lncRNAs play critical roles in the modulation of heart development and cardiovascular diseases (Wang et al., 2014, 2015; Zhang et al., 2016, 2017). For example, Shao et al. (2017) demonstrated that the expression of long noncoding RNAs TINCR was downregulated in the heart after a transverse aortic constriction (TAC) model (Shao et al., 2017). More recently, the same group showed that TINCR could epigenetically inhibit the transcription of CaMKII inhibiting cardiac hypertrophy induced by angiotensin II (Shao et al., 2017).

The evidence in the literature suggests that CaMKII is a key molecule for understanding the physiology and physiopathology of cardiovascular diseases as well as a prominent target for new strategies of treatment.

## Conclusion

**FIGURE 2:**
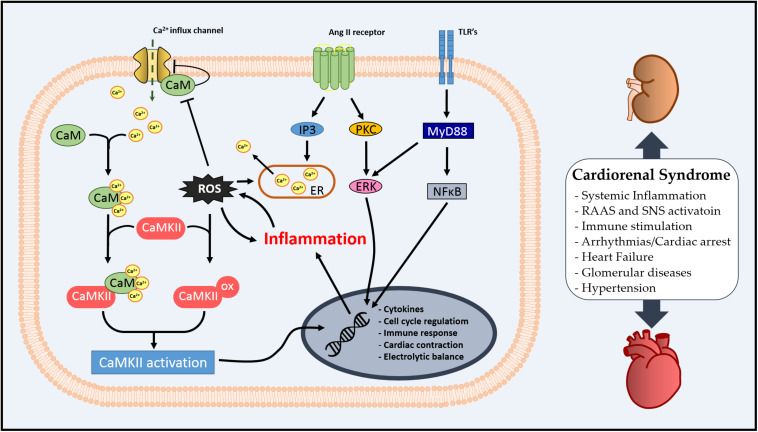
Schematic illustration of cellular Ca^2+^/calmodulin-dependent (CaMK) II involvement in cardiorenal syndrome (CRS).

In this review, we aimed to stimulate a discussion on the role of CaM as an inflammatory mediator in the systemic profile of CRS via regulation of CaMKII. The literature suggests that the Ca^2+^/CaM complex might be an important modulator of inflammation and oxidative stress via CaMKII in the kidney–heart interaction (summarized in Figure 2), and CaMKII regulation by pre- or post-translational mechanisms is essential for cardiac or renal homeostasis. Additionally, we have observed that CaM/CaMKII has been extensively studied in cardiovascular diseases; however, there is still the necessity of exploring how CaM could integrate Ca^2+^ signal in different scenarios, such as CRS.

## Author Contributions

CJ and MC-R proposed the idea and writing. All authors contributed to the article and approved the submitted version.

## Conflict of Interest

The authors declare that the research was conducted in the absence of any commercial or financial relationships that could be construed as a potential conflict of interest.

## Footnotes

1 Centers for Disease Control and Prevention. National Center for Health Statistics. “Leading Causes of Death: Deaths: Leading Causes for 2017, table 1. Available at: http://www.cdc.gov/nchs/fastats/leading-causes-of-death.htm [Accessed March 14, 2020].
